# Anaesthetic management of an infant with MEGD(H)EL syndrome undergoing cochlear implant

**DOI:** 10.1186/s12871-024-02812-2

**Published:** 2024-11-26

**Authors:** Nashwa Ahmed

**Affiliations:** Lecturer of Anaesthesia and Surgical Intensive Care, Faculty of Medicine, Port Said University, Port Fuad, Egypt

**Keywords:** MEGDEL syndrome, Dexmedetomidine, Ketamine, SERAC1 defect, Mitochondrial disease, Leigh-like

## Abstract

**Background:**

The syndrome has these features: 3-methylglutaconic aciduria (MEG), deafness(D), encephalopathy (E), Leigh-like syndrome (L). This disorder is caused by biallelic mutations in serine active site-containing protein 1 (SERAC1) gene. When these patients experience hepatopathy (H) in addition to the above manifestations, the syndrome is referred to as MEGD(H)EL. The pathology of this syndrome shares features with diverse types of inborn errors of metabolism.

**Case presentation:**

We discussed the anaesthetic management of an infant 2-year-old suffering from MEGD(H)EL syndrome undergoing cochlear implant. We discuss the pathology, genetics and significant aspects of this sporadic disease which is important for anaesthesiologist.

**Conclusions:**

The usage of dexmedetomidine as the main anaesthetic drug might have the benefit of a non-triggering anaesthetic agent in patients with a mitochondrial disease. Mixture of dexmedetomidine and ketamine provide an effective combination for procedural sedation, predominantly in select populations who are at a high risk of perioperative complications due to underlying co-morbid conditions.

## Introduction

The discovery of new congenital disorders due to advances in medicine. This puts the anaesthesiologists in situations to deal with newly recognized syndromes patients that are not familiar with them.

The syndrome has these features: 3-methylglutaconic aciduria (MEG), deafness(D), encephalopathy (E), Leigh-like syndrome (L). This disorder is caused by biallelic mutations in serine active site-containing protein 1 (SERAC1) gene. When these patients experience hepatopathy (H) in addition to the above manifestations, the syndrome is referred to as MEGD(H)EL [1]. 

We discuss the pathology, genetics and significant aspects of this sporadic disease which is important for anaesthesiologist [2]. 

Anaesthesiologists often encountered mitochondrial disease patients in the theatre. This disease is presented with clinical symptoms and signs in major organs, such as the kidneys, lungs, brain and liver which consume a lot of ATP. Mitochondria is the main source of metabolism in humans [3].

So, mitochondrial syndrome is hectic for the anaesthesiologist because mitochondrial disease causes dysfunctions of multiple organs, including cardiorespiratory failure, and myopathy [3]. 

Anaesthetic agents’ selection is crucial because significant and unexpected complications can happen after anaesthesia, although various anaesthetic methods have been effectively employed for those patients [3]. 

### Mitochondrial overview

Mitochondrial structure consists of four parts: the external membrane, the internal membrane, the intermembrane space, and the matrix. They do tasks, such as Krebs cycle, pyruvate oxidation, and the metabolism of amino acids, fatty acids, and steroids, nevertheless, the most important is energy creation as adenosine triphosphate (ATP), through the electron-transport chain and the oxidative-phosphorylation system (the “respiratory chain”). Mitochondria is the principal source of cell metabolism in humans. The cellular machinery necessary for the Krebs cycle, metabolism of amino acids, fatty acid oxidation and, most importantly, oxidative phosphorylation all exist within mitochondria, either in the mitochondrial matrix or mitochondrial membrane. Electrons usually join the electron transport chain via complex I or complex II and are then sequentially transferred to Coenzyme Q, complex III, cytochrome c, complex IV and finally to oxygen to form water [4, 5]. 

The energy regained during this transfer is employed to pump protons into the intermembrane space of the mitochondria, generating a gradient across the inner mitochondrial membrane. The proton gradient is then used as an energy source for phosphorylation of ADP to ATP by complex V [6]. 

The entire process is termed oxidative phosphorylation, and the complete system is termed the mitochondrial respiratory chain (MRC) (complexes I-V).

The respiratory chain is made up of five multiple protein complexes that are situated in the inner mitochondrial membrane: reduced nicotinamide adenine dinucleotide (NADH) dehydrogenase-ubiquinone oxidoreductase (complex I, approximately 46 subunits), succinate dehydrogenase-ubiquinone oxidoreductase (complex II, 4 subunits), ubiquinone-cytochrome c oxidoreductase (complex III, 11 subunits), Cytochrome c oxidase (complex IV, 13 subunits), and ATP synthase (complex V, approximately 16 subunits). Ubiquinone (coenzyme Q10) and cytochrome c are two minor electron carriers that are essential for the respiratory chain [5]. 

Two coordinated processes take part in ATP synthesis, with electrons transported along complexes to molecular oxygen and producing water. Protons shifted from the matrix to the intermembrane space by complexes I, III, and IV simultaneously [5]. 

### Genetics

Mitochondria are the only organelles that have their own DNA (mitochondrial DNA or mtDNA), and their own cellular machinery for producing RNA and protein. Since all mitochondria are maternally inherited, mutations in mtDNA are passed on to all offspring of an affected mother, but only transmitted further through her daughters [6]. 

Mitochondrial DNA codes only 37 genes (13 proteins, 22 transfer RNAs, 2 ribosomal RNAs); the other1100 or so gene products within the organelle are encoded by genes within the nuclear genome of the cell [4, 5]. and thus follow classical Mendelian inheritance patterns. Furthermore, mitochondria are under dual genetic control (Mendelian and mitochondria) [6]. 

Due to this dual genetic control, mitochondrial deficiencies may arise from many genetic causes, with different physiological presentations and modes of inheritance. Consequently, different offspring from a single mother may show strong variation in phenotype despite being genetically similar. So, it would be unsuitable to conclude that a drug or anaesthetic method being used safely with one patient having a mitochondrial defect would be similarly safe in all other patients with mitochondrial disease even in siblings with genetic variations resulting in identical mutations [6]. 

### Mitochondrial disease and cochlear implant

It is merit repeating that mitochondrial disease is not one disorder but stands for hundreds of various enzymatic mitochondrial defects, both genetic and environmental in origin [7]. 

Clinical symptoms alone are not pathognomonic in mitochondrial disease, and although radiological and laboratory investigations give important clues, muscle biopsy stays a crucial part of the diagnostic process [8]. 

Mutations in the serine active site-containing protein 1 (SERAC1) gene cause MEGDEL syndrome [3-methylglutaconic aciduria (MEG), deafness (D), encephalopathy (E), Leigh-like syndrome (L). (H) is added to the above manifestations When those patients experience hepatopathy, the syndrome is labelled as MEGD(H)EL [2]. 

Wortmann et al., in 2006 describe the serine active site-containing protein 1 which codes a protein important for the transformation of monoacylglycerol phosphate, and phospholipid phosphatidylglycerol that are crucial for function of the mitochondrial and cholesterol metabolism [2]. 

This syndrome is presented by severe dystonia, deafness, seizures spasticity, failure to thrive, and delayed developmental milestones. About 50% of those with neonatal onset have hepatic involvement ranging from severely abnormal liver enzymes, direct hyperbilirubinemia, and hyperammonia to severe liver dysfunction, however this is usually transient and occurs mostly through the first year of life [9]. 

MEGD[H]EL syndrome shares manifestations which are related to organic acidurias, the SERAC1 gene mutation also impairs oxidative phosphorylation, a mechanism analogous to mitochondrial disease. Yet, this syndrome is unique [10].

This syndrome had mentioned in many literatures and is predictable worldwide to happen in 27 births annually with a male: female ratio of 1:1.3 [9, 11] and the survival is approximately 50% in the teenage group [9] There is no. treatment only supportive. They may need several interventions that require anaesthesia, for example, sedation for auditory brainstem response testing, gastrostomy tube placement and magnetic resonance imaging.

In recent times, no clinical studies describe the consequences perioperatively of this syndrome. Furthermore, familiarity with anaesthetic management for patients suffering from mitochondrial disease and organic aciduria, can be guided to put effective plan for of these patients [2] 

Hearing loss has been seen often in those patients, However, due to life threatening features of this syndrome, there were fewer studies on the type and degree of severity of hearing loss in this disease. Gold and Rapin conducted a study in 1994, on correlation between deafness and mitochondrial disease, citing 32 from total 162 had hearing loss [12]. 

Cochlear implantation has been increasing rapidly, and now it is an efficient option for patients with severe hearing loss and deaf mutism. It had been one of the biggest advancements in otology. Cochlear implants are highly priced computerized electric prostheses that partially substitute for the functions of the cochlea [13].

The surgery is done under general anaesthesia through a trans-mastoid approach. The operative procedure requires the preservation of functional integrity of the facial and cochlear nerve. The anaesthesiologist is an integral part of the cochlear implant crew whose anaesthetic as well as communication skills are evaluated [13]. 

The anaesthesia technique plays a significant role in accomplishment of cochlear implant surgery as the anesthesiologist must produce circumstances which ease usage of nerve stimulators and management of post-operative complications such as nausea, vomiting and vertigo [13]. 

In recent years, no case reports or clinical trials are referring to the consequences perioperatively of this syndrome.

Additionally, familiarity with anaesthetic management of patients with mitochondrial disorders, organic acidurias, and fatty acid metabolism can provide insights and contribute to creating a plan that efficiently addresses potential difficulties.

### Case presentation

We obtained written informed consent from the patient parents to publish this case report. We discuss the anaesthetic plan of a female diagnosed to be MEGD(HE)L syndrome aged 2 years, weighing 8 kg, scheduled for cochlear implant under general anaesthesia.

Full term baby had been born through the caesarean section due to abnormal presentation and her weight was 3200 g. Full investigations and work-up were done showed generalized muscular hypotonia elevated liver enzymes, hyperbilirubinemia, lactic acidosis, hypoglycemia and hyperammonemia.

She had been identified by genetic testing to have MEGD(H)EL syndrome. After adjustment of the metabolic abnormalities, she was released home on day 14 postnatal.

On physical examination revealed a grossly normal and age-appropriate airway with generalized hypotonia. She is currently taking l-carnitine, vitamin E, and coenzyme Q-10.

Built on existing data, there is no therapeutic treatment for this developing disease [2]. She experienced a generalized delay in developmental milestones, and a profound bilateral hearing loss. No evidence of seizure disorder existed. No previous exposure to anaesthesia.

A careful anaesthesia protocol was outlined, we decided to use infusion of dexmedetomidine for maintenance of sedation during the surgery. The anaesthesia machine had been prepared by a 60-minute highflow gas flush at 10 1. minute^− 1^, removing the vapourisers, and replacing the soda lime. On the morning of the surgery, she was allowed in the theatre with intravenous access in place and was fasting for at least 4 h.

Intravenous (IV) dextrose 5% at the rate of 25 ml. hour^− 1^ was started 2 h before the surgery as maintenance fluid and continued till the anaesthesia induction. Preinduction glucose level was 70 mg/dl in blood.

After preoxygenation, induction of anaesthesia intravenous with ketamine 2 mg.kg^− 1^ fentanyl 1 µg.kg^− 1^ and dexmedetomidine 1 µg.kg^− 1^. Intubation was done using cuffed tube of appropriate size. Anaesthesia maintenance had been done with IV infusion of dexmedetomidine at the rate of 0.5 µg.kg^− 1^. hour^− 1^. Intraoperative facial nerve monitoring was performed. The starting temperature was 36.4^0^C and the infant was cautiously warmed by a heating mattress. The surgery period was uneventful and was completed within 90 min.

The haemodynamic and saturation had been checked and were within normal ranges. At the surgery end, brainstem evoked response audiometry (BERA) is employed to verify the integrity of the implant. Dexmedetomidine infusion was stopped at the end of procedure, and she was observed for recovery from anaesthesia, glucose level, vital signs and temperature.

After 10 min, she recovered from anaesthesia and the hemodynamics were stable and the temperature was 36.6^0^C. The temperature was measured pre and post operative rectally. Analgesia was achieved postoperatively with rectal suppositories of paracetamol. The patient’s postoperative course and recovery were uneventful with adequate pain management. The patient was shifted for observation postoperatively to the ICU for the first 24 h [14]. 

The ICU period was smooth, and she was released the next day. The anaesthetic plan was tolerated without any complications.

In our literature, we would like to focus on the of dexmedetomidine usage as sole anaesthetic agent in MEGDEL syndrome patients. Dexmedetomidine had been employed as an alternative for anaesthetic drugs, to prevent mitochondrial dysfunction and malignant hyperthermia.

We also aimed to avoid any metabolic abnormalities at the perioperative and postoperative period, which includes hypoglycemia, hypovolemia and hypothermia [14, 15]. 

## Discussion

To our information, our case report is the first to explain the anaesthetic management of a patient with MEGD(H)EL syndrome, a new, and rare congenital metabolic disorder undergoing cochlear implant.

Anaesthetic management of patients with mitochondrial disease demands meticulous care for cardiorespiratory system and precise attention to mitochondrial function. Patients suffering from hypotonia, and generalized myopathy might be susceptible to upper airway obstruction intra and postoperative, hypercarbia, hypoventilation, and acidosis [7]. 

They also benefit from opioids sparing techniques such as the usage of regional anaesthesia, local anaesthetics, NSAIDs, and avoidance of nondepolarizing neuromuscular blocking drugs to which patients with mitochondrial disease might have excessive sensitivity. Depolarizing muscle relaxants as succinylcholine must not be administered in any patient with myopathy due to their upregulation of nicotinic acetylcholine receptors in skeletal muscle, resulting in potentially fatal acute hyperkalemia and acute myolysis after succinylcholine administration [7]. 

In the perioperative setting, several measures are taken to reduce physiologic changes and thus stress impaired mitochondrial function. A full anaesthetic assessment must be conducted to figure out baseline comorbidities and the extent of organ system involvement. In particular, the history and physicians must ask for symptoms and signs of mitochondrial disease, such as cardiomyopathy, respiratory weakness, obstructive sleep apnea, seizures, and lactic acidosis. Preoperative fasting is reduced to 2 h, if possible, to avoid hypovolemia and hypoglycemia [7]. 

Dextrose-containing fluids have to be utilized for all fasting patients with the following exception, patients with mitochondrial disease on ketogenic diets for control of seizure [16]. When patients receive dextrose, they should be checked for any hyperglycemia or lactic acidosis [17]. Continuous temperature monitoring is employed to avoid both hypo- and hyperthermia, with the usage of active patient warming or cooling devices, and intravenous fluid warmers to maintain euthermia [7]. 

Continuous intraoperative electrocardiogram, non-invasive blood pressure, and end-tidal gas monitoring are essential for all patients undergoing surgery. Maintenance anaesthetics must be monitored gradually, slowly, and cautiously, while checking anaesthetic depth clinically or ideally with the BIS or other processed electroencephalography system if available [7]. 

It is crucial to memorize that some patients with mitochondrial disease have shown remarkable sensitivity to anaesthetic agents [18]. Although induction of general anaesthesia with either propofol bolus or volatile anaesthetics has been used in patients with mitochondrial disease. It is recommended to refrain from administering propofol by continuous infusion after induction of anaesthesia due to concerns about propofol infusion syndrome in this vulnerable population. However, this case report and other studies believe that boluses of propofol are well tolerated unless the patient is metabolically disturbed [7]. 

The following had been used safely to give anaesthesia without triggering mitochondrial decompensation such as, small intravenous boluses of propofol, benzodiazepines, or ketamine; continuous infusion of dexmedetomidine, inhalation of sevoflurane; and bolus dosing or continuous infusion of short- or ultrashort-acting opioids such as fentanyl, sufentanil, alfentanil, or remifentanil [7]. 

Volume replacement with a physiological electrolyte solution without lactate is preferable since patients with mitochondrial disease might have impaired lactate metabolism. The question of how long to keep a patient with mitochondrial disease under observation postoperatively usually revolves around how long the patient is likely to be in a catabolic state [7]. 

Close postoperative monitoring is necessary to guarantee that patients restore their baseline level of function prior to discharge from the post anaesthesia unit [7]

About mitochondrial disorder, many anaesthetic drugs hinder function of mitochondrial [19, 20]. Even though, nearly all agents had been effectively employed in these patients when the safety measures were applied [7, 21]. ketamine where a doubt exists, the solitary exemptions are dexmedetomidine without impairment of mitochondrial function and [2]. 

Horvath et al. had studied the intravenous mixture of dexmedetomidine and midazolam infusion for sedation patients scheduled for the MRI scan [2]. 

Low-dose midazolam has been used effectively in mitochondrial disease patients. It had been reported that dexmedetomidine no significant consequences on function of the mitochondria [7]. Furthermore, it was reported that dexmedetomidine possesses a protecting effect on mitochondria in an animal model [22]. These aspects made dexmedetomidine a suitable drug for anaesthesia in this syndrome and other mitochondrial diseases [2]. 

We had selected dexmedetomidine for anaesthesia for avoidance of the potential side effects of inhaled anaesthetics and propofol. There was no evidence of the side effects of dexmedetomidine in mitochondrial disease.

In mitochondrial disease, successful clinical studies had been done using non-triggering anaesthesia with dexmedetomidine. Dexmedetomidine effect on nervous system is unlike other intravenous anaesthetics, it acts on a2 adrenoceptors in the locus coeruleus [3]. 

Dexmedetomidine usage avoids the underlying mechanism that impair mitochondrial function. It also has reduced depression of respiration, which may occur during recovery of anaesthesia, this could be another advantage. A new clinical trial revealed that it had decreased the damage accompanying with mitochondrial stress [23].

It alleviated nervous system insult caused by mitochondrial disease. Nevertheless, there are multiple restrictions to the usage of the anaesthetic method in this case report. dexmedetomidine alone is still a sedative analgesic drug and there had been limited literature about the usage as the sole anaesthetic agent [3]. 

However, a new study presented that dexmedetomidine could be a sole anaesthetic agent, it was used as solitary anaesthetic drug during laparotomy surgery given by bolus injection 1 µg.kg-1 and then by infusion (0.5 µg.kg^− 1^.h^− 1^) for anaesthesia maintenance [3]. 

Further research and clinical trials are required to study the use of dexmedetomidine as a sole anaesthetic. Despite these limitations, the usage of dexmedetomidine as the main anaesthetic drug might have the benefit of a non-triggering anaesthetic agent in patients with a mitochondrial disease.

A lot of the trials revealed that dexmedetomidine avoid increase in heart rate and blood pressure, salivation and emergence phenomena that occur with ketamine and in turn ketamine, prevented occurrence of hypotension and bradycardia caused with dexmedetomidine. The mixture of both drugs was recognized as a secure combination [24]. 

A mixture of dexmedetomidine and ketamine were used as the primary anaesthetic, with each counteracting the unfavorable hemodynamic effects of the other. Our practice and previous studies from the literature recommend the mixture of dexmedetomidine and ketamine provide an effective combination for procedural sedation, predominantly in select populations who are at a high risk of perioperative complications due to underlying co-morbid conditions [25]. 

## Conclusions

Pediatric anaesthesiologists must be familiar with the disease as patients’ number suffering from MEGD(H)EL syndrome is predictable to increase.

The anaesthetic techniques and drugs choice, fluid management must be created upon the understanding of the pathology of disease.

Hypoglycemia is the most common symptom in the neonatal period, so the level of glucose must be checked and maintained with supplement of glucose infusions.

Centrally acting alpha-adrenergic agonists such as dexmedetomidine can be employed for sedation as an alternative to propofol. Dexmedetomidine don’t cause respiration depression, protects airway reflexes, provides excellent anxiolysis and sedation. It had been reported to be safe in mitochondrial disease since the mechanism of action in the central nervous system is different from often used general anaesthetic agents. Successful trials had been done using non-triggering anaesthesia with dexmedetomidine in mitochondrial disease.

Further prospective studies will enable a thorough analysis of perioperative patient management and the usage of general anaesthesia in mitochondrial disease, with the aim of establishing evidence-based clinical guidelines.

## Data Availability

The raw data supporting the conclusions of this article will be made availableby the corresponding author. No datasets were generated or analyzed during the current study.

## Abbreviations

SERAC1: Serine active site-containing protein 1 gene
NSAIDs: Nonsteroidal anti-inflammatory drug
MRI: Magnetic Resonance Imaging
